# Phylogenetic significance of composition and crystal morphology of magnetosome minerals

**DOI:** 10.3389/fmicb.2013.00344

**Published:** 2013-11-26

**Authors:** Mihály Pósfai, Christopher T. Lefèvre, Denis Trubitsyn, Dennis A. Bazylinski, Richard B. Frankel

**Affiliations:** ^1^Department of Earth and Environmental Sciences, University of PannoniaVeszprém, Hungary; ^2^Laboratoire de Bioénergétique Cellulaire, Biologie Végétale et Microbiologie Environnementales, CEA/CNRS/Aix-Marseille UniversitéSaint Paul lez Durance, France; ^3^School of Life Sciences, University of Nevada at Las VegasLas Vegas, NV, USA; ^4^Department of Physics, California Polytechnic State UniversitySan Luis Obispo, CA, USA

**Keywords:** magnetotactic bacteria, magnetite, greigite, magnetosomes, morphology, biomineralization, evolution

## Abstract

Magnetotactic bacteria (MTB) biomineralize magnetosomes, nano-scale crystals of magnetite or greigite in membrane enclosures that comprise a permanent magnetic dipole in each cell. MTB control the mineral composition, habit, size, and crystallographic orientation of the magnetosomes, as well as their arrangement within the cell. Studies involving magnetosomes that contain mineral and biological phases require multidisciplinary efforts. Here we use crystallographic, genomic and phylogenetic perspectives to review the correlations between magnetosome mineral habits and the phylogenetic affiliations of MTB, and show that these correlations have important implications for the evolution of magnetosome synthesis, and thus magnetotaxis.

## Introduction

All magnetotactic bacteria (MTB) contain magnetosomes comprising nano-scale, magnetite (Fe_3_O_4_) or greigite (Fe_3_S_4_) crystals enclosed in phospholipid bilayer membranes (Gorby et al., 1988; Bazylinski and Frankel, 2004). The magnetosomes constitute a permanent magnetic dipole moment in the cell, and are essential for magnetotaxis. The magnetosome membrane is derived by invagination of the cytoplasmic membrane (Komeili et al., 2004) and is the locus of biological control over the nucleation and growth of the mineral crystal. Most MTB species or strains exclusively produce either magnetite (Frankel et al., 1979) or greigite magnetosomes (Mann et al., 1990), although several MTB can produce magnetosomes of both kinds, depending on environmental conditions (Bazylinski et al., 1993; Kasama et al., 2006; Lins et al., 2007; Lefèvre et al., 2011c; Wang et al., 2013).

The crystal size, crystallographic orientation and arrangement of magnetosomes in MTB are all highly significant for the magnetic properties of the cell (Frankel and Blakemore, 1980; Mann et al., 1984a,b; Moskowitz et al., 1988; Bazylinski and Frankel, 2003). With a few exceptions, the lengths of individual magnetosome crystals range from about 35 to 120 nm (Devouard et al., 1998) (Table 1); this is within the permanent single-magnetic-domain (SD) size range for both minerals (Butler and Banerjee, 1975). In the majority of MTB, the magnetosomes are organized in one or more straight chains of various lengths, parallel to the axis of motility of the cell. In cells of some species, however, there are multiple individual chains or a chain with multiple strands (Vali and Kirschvink, 1991) or even dispersed aggregates or clusters of magnetosomes that occur in some magnetotactic cocci (Towe and Moench, 1981; Cox et al., 2002; Zhang et al., 2012).

**Table 1:** **Bibliographic listing of magnetotactic bacteria characterized and the composition and morphology of their magnetosome crystals analyzed**.

**Magnetosome mineral**	**Strain**	**Phylogenetic affiliation**	**Habit**	**Magnetosome elongation axis**	TEM technique of morphology determination^*^	**Average crystal length (nm)**	**Shape factor (width/length)**	**References**
**Magnetite**	*Magnetospirillum magnetotacticum* strain MS-1	*Alpha-proteobacteria*	cuboctahedral	^**^	Single-projection BF, SAED, HRTEM, EH, BF ET	43	0.9	Devouard et al., 1998; Buseck et al., 2001; Kobayashi et al., 2006
**Magnetite**	*Magnetospirillum magneticum* strain AMB-1	*Alpha-proteobacteria*	cuboctahedral	^**^	Single-projection BF, SAED, HRTEM	45	0.85	Li et al., 2009
**Magnetite**	*Magnetospirillum gryphiswaldense* strain MSR-1	*Alpha-proteobacteria*	cuboctahedral	^**^	Multi-projection BF, SAED, HRTEM, BF ET	33	0.91	Scheffel et al., 2006; Faivre et al., 2008
**Magnetite**	*Magnetospira thiophila* strain MMS-1 (MV-4)	*Alpha-proteobacteria*	elongated, octahedral	[111]	Single-projection BF, SAED, HRTEM	22–85	0.85	Meldrum et al., 1993a; Devouard et al., 1998
**Magnetite**	*Magnetovibrio blakemorei* strain MV-1	*Alpha-proteobacteria*	elongated, octahedral	[111]	Multi-projection BF, SAED, HRTEM, HAADF ET	60	0.65	Meldrum et al., 1993a; Devouard et al., 1998; Thomas-Keprta et al., 2001; Clemett et al., 2002
**Magnetite**	*Magnetovibrio blakemorei* strain MV-2	*Alpha-proteobacteria*	elongated, prismatic	[111]	Single-projection BF, SAED, HRTEM	30–59	0.54	Meldrum et al., 1993a
**Magnetite**	*Magnetococcus marinus* strain MC-1	*Alpha-proteobacteria*	octahedral, elongated	[111]	Single-projection BF	30–110	0.93	Meldrum et al., 1993b; Devouard et al., 1998
**Magnetite**	Strain MC-2	*Alpha-proteobacteria*	octahedral, elongated	ND	Single-projection BF, SAED, HRTEM	30–120	0.85	Devouard et al., 1998
**Magnetite**	*Candidatus* Magnetococus yuandaducum strain YDC-1	*Alpha-proteobacteria*	elongated, prismatic	ND	Single-projection BF	108	0.64	Lin and Pan, 2009
**Magnetite**	Strain MO-1	*Alpha-proteobacteria*	octahedral, elongated	ND	Single-projection BF	64	0.89	Lefèvre et al., 2009
**Magnetite**	*Magnetospira* sp. QH-2	*Alpha-proteobacteria*	octahedral, elongated	ND	Single-projection BF	81	0.71	Zhu et al., 2010
**Magnetite**	uncultured coccus Itaipu-I	ND	elongated, prismatic	[111]	Multi-projection BF, SAED, HRTEM, EH	210	0.9	Lins et al., 2005
**Magnetite**	uncultured coccus Itaipu-III	ND	elongated, prismatic	[111]	Multi-projection BF, SAED, HRTEM, EH	130	0.6	Lins et al., 2005
**Magnetite**	uncultured coccus	ND	elongated, prismatic	[111]	Multi-projection BF, SAED, HRTEM, HAADF ET	< 80	0.88	Simpson et al., 2005
**Magnetite**	uncultured coccus	ND	elongated, prismatic	ND	HAADF ET	ND	ND	Buseck et al., 2001
**Magnetite**	Strain BW-2	*Gamma-proteobacteria*	octahedral	ND	Single-projection BF	67	0.94	Lefèvre et al., 2012
**Magnetite**	Strain SS-5	*Gamma-proteobacteria*	octahedral, elongated	[111]	Single-projection BF, SAED, HRTEM	86	0.75	Lefèvre et al., 2012
**Magnetite**	Strain ZZ-1	*Delta-proteobacteria*	elongated, bullet, dts^1^	ND	Single-projection BF	84^***^	0.44^***^	Lefèvre et al., 2011b
**Magnetite**	Strain ML-1	*Delta-proteobacteria*	elongated, bullet, dts^1^	ND	Single-projection BF	ND	ND	Lefèvre et al., 2011b
**Magnetite**	Strain AV-1	*Delta-proteobacteria*	elongated, bullet, dts^1^	[100]	Multi-projection BF, SAED, HRTEM	30–120	0.45	Lefèvre et al., 2011d
**Magnetite**	*Desulfovibrio magneticus* strain RS-1	*Delta-proteobacteria*	elongated, bullet	[100]	Multi-projection BF, SAED, HRTEM, BF ET	40	0.5	Sakaguchi et al., 1993; Pósfai et al., 2006
**Magnetite**	*Ca*. Desulfamplus magnetomortis strain BW-1	*Delta-proteobacteria*	elongated, bullet	ND	Multi-projection BF, SAED, HRTEM	55^***^	0.6^***^	Lefèvre et al., 2011c
**Magnetite**	Uncultured Multicellular	*Delta-proteobacteria*	elongated, bullet, dts^1^	[100]	Single-projection BF, SAED, HRTEM	104	0.4	Keim et al., 2007
**Magnetite**	*Ca*. Magnetananas tsingtaoensis	*Delta-proteobacteria*	elongated, bullet	ND	Single-projection BF	102	0.37	Zhou et al., 2012
**Magnetite**	*Ca*. Magnetobacterium bavaricum	*Nitrospirae*	elongated, bullet	ND	Single-projection BF	110–150	ND	Spring et al., 1993
**Magnetite**	Strain MHB-1	*Nitrospirae*	elongated, bullet	ND	Single-projection BF	119^***^	0.35^***^	Flies et al., 2005
**Magnetite**	Strain MYR-1	*Nitrospirae*	elongated, bullet	[100]	Multi-projection BF, SAED, HRTEM	104	0.36	Li et al., 2010
**Magnetite**	Strain MWB-1	*Nitrospirae*	elongated, bullet	ND	Single-projection BF	116	0.35	Lin et al., 2012
**Magnetite**	*Ca*. Magnetoovum mohavensis strain LO-1	*Nitrospirae*	elongated, bullet, fts^2^	[110]	Multi-projection BF, SAED, HRTEM	70–200	0.36	Lefèvre et al., 2011a,d
**Magnetite**	*Ca*. Thermomagnetovibrio paiutensis strain HSMV-1	*Nitrospirae*	elongated, bullet, fts^2^	[110]	Multi-projection BF, SAED, HRTEM	30–220	0.45	Lefèvre et al., 2010, 2011d
**Magnetite**	Strain SKK-01	Candidate division OP3	elongated, bullet	ND	Single-projection BF	110	0.34	Kolinko et al., 2012
**Greigite**	Uncultured MMP	*Delta-proteobacteria*	equidimensional, irregular; elongated, irregular	^**^ and [100]	Multi-projection BF, SAED, HRTEM	60–90	0.86^***^	Pósfai et al., 1998a,b
**Greigite**	*Ca*. Desulfamplus magnetomortis strain BW-1	*Delta-proteobacteria*	equidimensional, irregular	ND	Multi-projection BF, SAED, HRTEM	33^***^	0.96^***^	Lefèvre et al., 2011c
**Greigite**	Uncultured rods	ND	equidimensional, irregular	^**^ and [100]	HAADF ET	60	0.9	Kasama et al., 2006
**Greigite**	*Ca*. Magnetomorum litorale	*Delta-proteobacteria*	elongated, bullet	ND	Single-projection BF	91	0.44	Wenter et al., 2009

* BF, bright-field

SAED, selected-area electron diffraction; HRTEM, high-resolution transmission electron microscopy; EH, electron holography; ET, electron tomography; HAADF, high-angle annular dark-field. ND, not determined.

** These crystals are equidimensional, therefore there is no elongation.

*** Estimated from published TEM micrographs in appropriate references.

1 dts, double triangle shape

2 fts, flat top shape.

When magnetosomes are arranged in chains, magnetic interactions between them cause their magnetic moments to orient parallel to each other along the chain axis (Frankel and Blakemore, 1980; Frankel, 1984), resulting in a permanent, magnetic dipole. The permanent magnetism of magnetosome chains has been demonstrated by electron holography in the electron microscope (Dunin-Borkowski et al., 1998), by pulsed magnetic field remanence measurements on individual cells (Penninga et al., 1995; Hanzlik et al., 2002) and by magnetic imaging directly in living cells (Le Sage et al., 2013).

The magnetosome membrane originates from the cytoplasmic membrane and contains unique proteins that are not present in the cytoplasmic or outer membranes (Komeili, 2012). These proteins, specific to MTB, are designated with the prefix Mam or Mms, although some are not found in every species of MTB. The Mms proteins in particular are present only in certain phylogenetic groups of MTB. While not all Mam proteins are found in the magnetosome membrane, all Mms proteins are. The Mam and Mms proteins are thought to be responsible for biomineralization of the magnetosome crystal, the organization of the magnetosome chain, and the crystallographic orientation of the individual magnetosomes with respect to the chain (Komeili, 2012). The roles of relatively few of the magnetosome membrane proteins have been elucidated (Jogler and Schüler, 2009; Murat et al., 2010; Lohsse et al., 2011; Uebe et al., 2011; Komeili, 2012).

All known MTB are phylogenetically affiliated with the *Alpha*-, *Delta*- or *Gammaproteobacteria* classes of the *Proteobacteria* phylum, the *Nitrospirae* phylum or the candidate division OP3 which is part of the *Planctomycetes*–*Verrucomicrobia*–*Chlamydiae* (PVC) bacterial superphylum (Lefèvre and Bazylinski, 2013) (Table 1). While magnetite-producing MTB occur in all five taxa, greigite-producing bacteria are restricted to a particular clade of sulfate-reducing bacteria in the *Deltaproteobacteria* (Lefèvre et al., 2011c; Lefèvre and Bazylinski, 2013).

**Figure 1:**
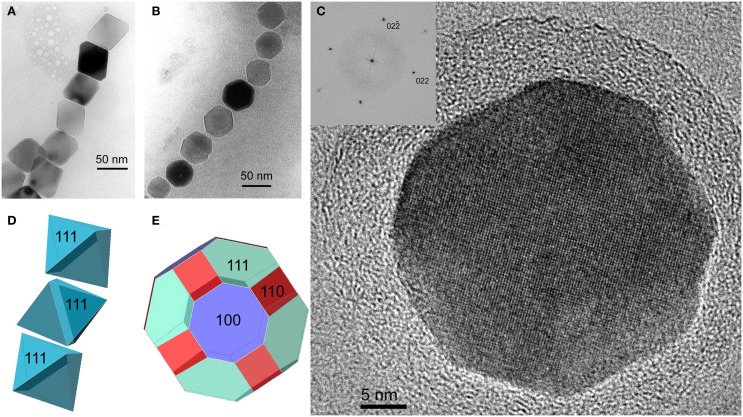
**Magnetite magnetosomes with octahedral and cuboctahedral morphologies. (A)** Transmission electron microscope (TEM) image of a partial chain of relatively regular octahedra in an unidentified freshwater spirillum. **(B)** TEM image of a partial chain of cuboctahedral magnetosomes in a cell of an alphaproteobacterial *Magnetospirillum* species isolated from Lake Ely, Pennsylvania. **(C)** High-resolution TEM image of a cuboctahedral magnetosome from the magnetotactic alphaproteobacterium *Magnetospirillum gryphiswaldense* strain MSR-1, with its Fourier transform inserted in the upper left, indicating that the crystal is viewed along the [100] direction. **(D)** Schematic model for a segment of the chain of octahedra in **(A)**. **(E)** A morphological model for the crystal shown in **(C)**; although the faces of the forms {111} (the octahedron) and {100} (the cube) dominate the morphology, smaller faces of {110} (the dodecahedron) also appear, resulting in an octagonal two-dimensional projection.

A compelling feature of magnetosome magnetite crystals is that they have species-specific, two-dimensional projected shapes when observed in an electron microscope (Figure 1). This implies that, in addition to size, orientation and arrangement, the magnetosome membrane proteins control the morphology of the magnetosome crystals.

In the past decade, a fortuitous confluence of advances in electron microscopy, increasing success in the axenic cultivation of MTB from diverse environments, and the availability of facilities for rapid sequencing of bacterial genomes, have revealed a relationship between magnetosome crystal composition and morphology and the phylogenetic affiliations of MTB. In this review we describe this relationship and also discuss the implications for the evolutionary history of magnetosome formation and magnetotaxis.

## Experimental determination of crystal morphology

Two-dimensional projections of magnetosomes in bright-field (BF) transmission electron microscopy (TEM) images have been used for the approximate evaluation of magnetosome morphologies (Matsuda et al., 1983; Mann et al., 1987a,b; Meldrum et al., 1993a, b; Devouard et al., 1998). However, without information about the thickness profile of each crystal, it is difficult to determine 3-dimensional (3D) habits from 2D images. For an unambiguous identification of magnetosome morphologies, it is necessary to tilt the specimen in order to obtain images along several projection directions (Pósfai et al., 2013). By taking into account constraints resulting from the known point group of magnetite, the morphologies of the crystals can be better interpreted and modeled (Lefèvre et al., 2011d). If multi-projection magnetosome outlines are complemented by selected-area electron diffraction (SAED) patterns and high-resolution (HR) TEM images obtained along certain crystallographic directions, the exact relationship between crystal morphology and internal structure can be established (Simpson et al., 2005; Pósfai et al., 2006; Faivre et al., 2008; Li et al., 2010; Lefèvre et al., 2011d).

The ultimate solution for obtaining the precise 3D morphologies of nanocrystals is provided by electron tomography (ET) (Pósfai et al., 2013). The technique is based on large numbers of images acquired as a function of specimen tilt angle, followed by 3D reconstruction and visualization. However, crystalline materials, including the minerals within magnetosomes, can exhibit strong diffraction contrast in BF TEM images. In such cases the intense diffracted beams are excluded from image formation, resulting in images in which the contrast is no longer dominated by variations in specimen thickness and density. A solution to this problem is provided by the acquisition of tilt series of high-angle annular dark-field (HAADF) images using a scanning transmission electron microscope (Midgley and Weyland, 2011). A HAADF detector collects electrons that are scattered at relatively large angles and are typically unaffected by the crystallography of the sample. Therefore, the contrast in HAADF images is directly related to the thickness of the material that the electron beam passed through, provided that the sample is homogeneous. HAADF ET has been used for the characterization of the morphologies of magnetite crystals from several strains of MTB (Table 1) (Buseck et al., 2001; Thomas-Keprta et al., 2001; Clemett et al., 2002; Kasama et al., 2006). A rarely used but possible alternative to ET is to obtain thickness information using electron holography for the reconstruction of 3D magnetosome morphologies (Lins et al., 2005).

## Magnetite magnetosome crystals

**Figure 2:**
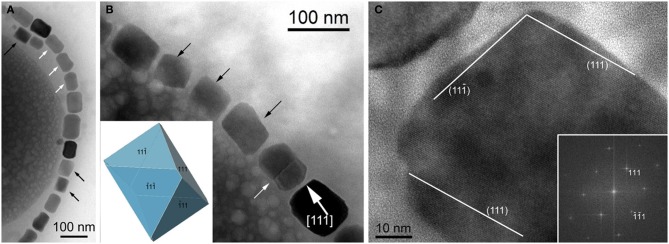
**Magnetite magnetosomes with elongated octahedral habits in the magnetotactic *Gammaproteobacteria* strain SS-5. (A)** TEM image of a chain of highly elongated magnetosomes. Black arrows mark crystals with pronounces octahedral facets, and white arrows point to magnetosomes with a “waisted” appearance, probably a result of twinning. **(B)** TEM image of part of a magnetosome chain with elongated octahedral habits (marked by black arrows and modeled in the lower left), a twinned crystal (marked by a white arrow), and a magnetosome showing slightly irregular surfaces, elongated approximately parallel to [111] (as indicated in the image). **(C)** High-resolution TEM image of the magnetosome in the lower right in **(B)**, viewed along [1–10], as indicated by the Fourier transform in the lower right. The surfaces of the crystal slightly deviate from the octahedral planes as marked in the image.

**Figure 3:**
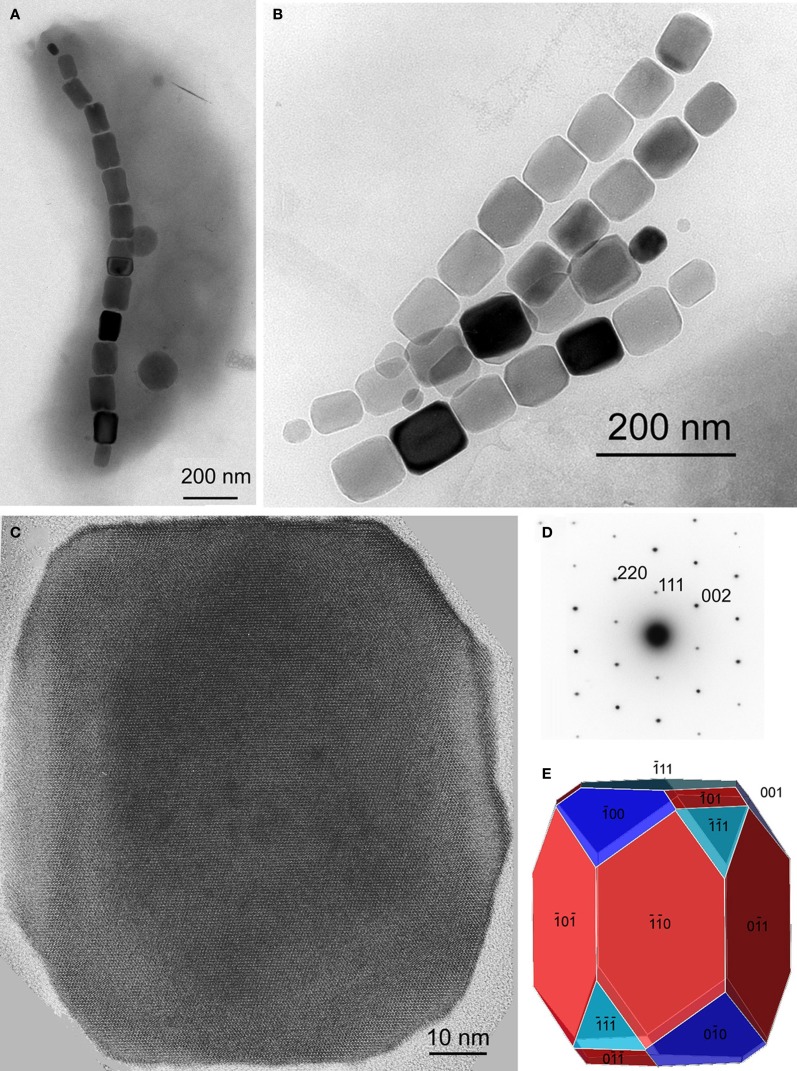
**Magnetite magnetosomes with elongated prismatic habits from magnetotactic *Alphaproteobacteria*. (A)** TEM image of a cell of a vibrioid MTB from Lake Mead, Nevada, containing a chain of elongated magnetosomes. **(B)** TEM image of two double chains of elongated magnetosomes from a freshwater coccus. **(C)** High-resolution TEM image of a magnetosome from a freshwater coccus with **(D)** its selected-area electron diffraction pattern (in [1–10] orientation) and **(E)** a morphological model that consists of six large and six small dodecahedral faces, and smaller faces of the cube and octahedron. The elongation direction is [111].

**Figure 4:**
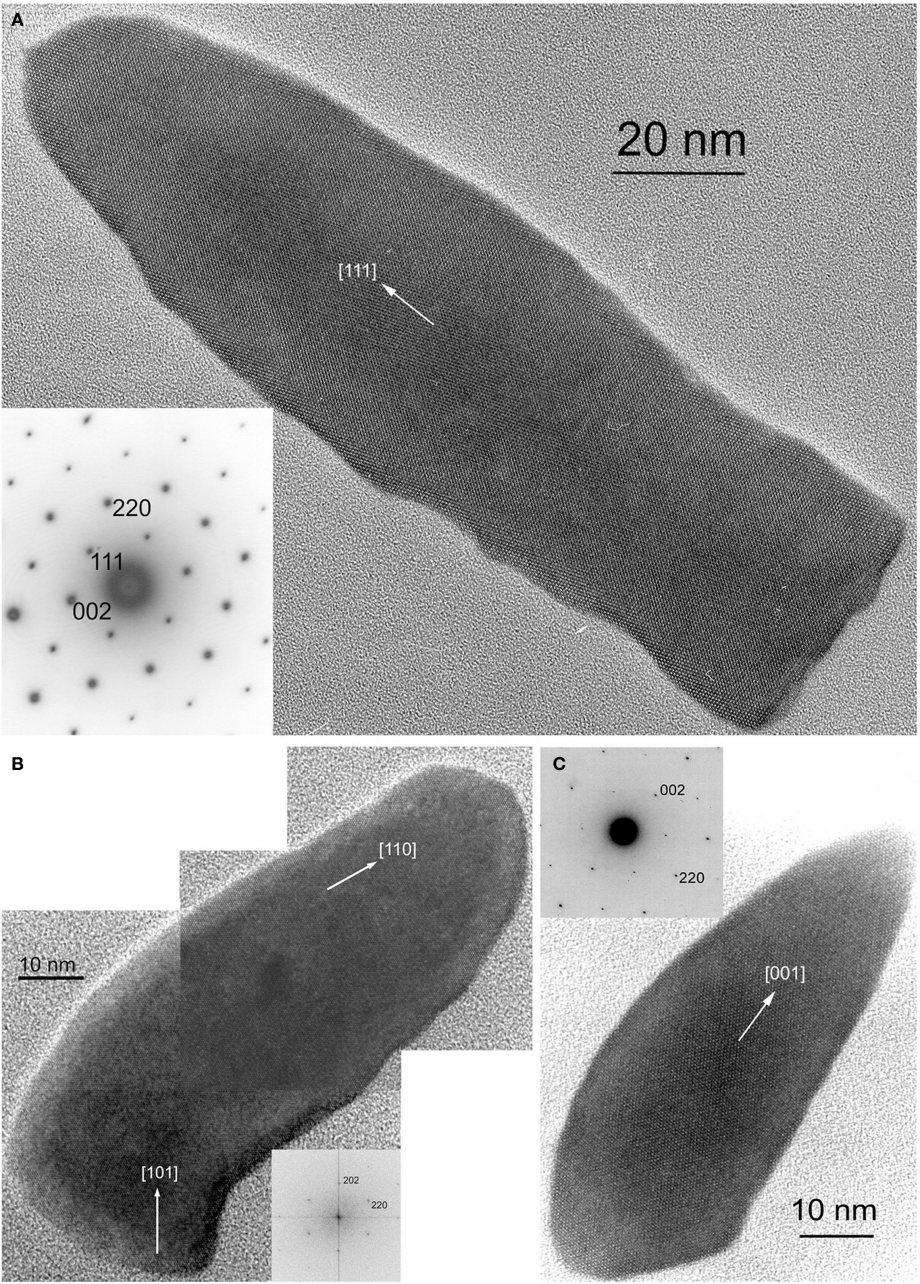
**HRTEM images of magnetite magnetosomes with highly anisotropic, elongated, pointed habits but different elongation directions. (A)** A magnetosome from an unidentified freshwater rod, elongated parallel to [111], with the corresponding selected area electron diffraction (SAED) pattern in the lower left. **(B)** A composite image of a curved, fts magnetosome from the magnetotactic *Nitrospirae* strain HSMV-1, elongated parallel to [110], with the corresponding Fourier transform in the lower right. **(C)** A dts magnetosome form the magnetotactic *Deltaproteobacteria* strain AV-1, elongated parallel to [001], with the corresponding SAED pattern in the upper left. All three images were obtained with the electron beam parallel to [1–10].

**Figure 5:**
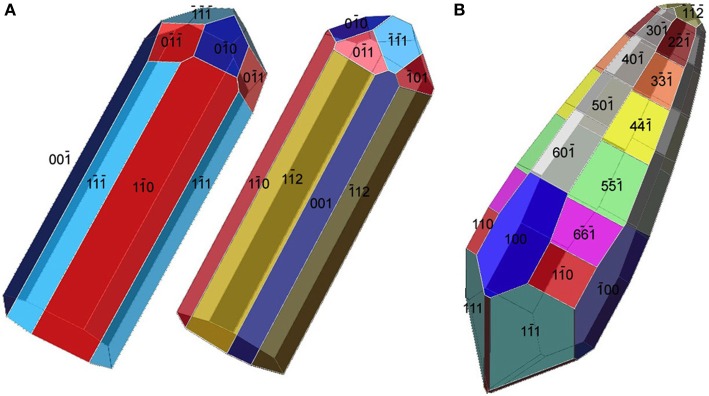
**Tentative morphological models for the elongated magnetosomes in Figures 4B,C. (A)** Two possible morphologies for the magnetosome in Figure 4B. The curving of the magnetosome is not taken into account. Both models are elongated along [110] but have different forms as their prismatic faces. **(B)** An approximate model for the morphology of the [001]-elongated magnetosome in Figure 4C.

The minerals magnetite and greigite are isostructural, with face-centered cubic, inverse-spinel crystal structures (Fd3m space group) (Palache et al., 1944). Three idealized habits based on the low-index forms {100}, {110}, and {111} have been described for magnetite crystals in magnetosomes. These include equidimensional [octahedra and cuboctahedra, a morphology with faces of the {100} (cube) and {111} (octahedron)]; elongated-prismatic; and elongated-anisotropic (Lefèvre et al., 2011d) (Table 1). The cuboctahedral crystal morphology, with six equivalent faces of the form {100} and eight equivalent faces of the form {111}, preserves the symmetry of the cubic crystal system and is considered close to the equilibrium growth form of magnetite (Mann and Frankel, 1989; Devouard et al., 1998) (Figure 1). Elongated octahedral habits also occur in some strains (Figure 2). The elongated-prismatic crystals are cuboctahedra with enhanced growth parallel to one of the < 111 > axes. This causes the differential growth of some symmetry-related crystal faces and introduces faces of the form {110} (Figure 3). The growth of the elongated-anisotropic crystals appears to be more complex because this habit lacks a center of symmetry that represents a greater departure from equilibrium (Mann and Frankel, 1989; Li et al., 2010; Lefèvre et al., 2011d). The elongated-anisotropic crystals typically have high-index faces in addition to those of the three low-index forms described above, and can be further grouped into several subcategories depending on their elongation directions (Figures 4, 5).

Magnetotactic *Alpha*- and *Gammaproteobacteria* mineralize magnetite magnetosome crystals with cuboctahedral, elongated octahedral or elongated prismatic habits (Figures 1–3) (Mann et al., 1984a; Lefèvre et al., 2012). For instance, it was shown that MTB of the genus *Magnetospirillum* in the *Alphaproteobacteria* mineralize magnetosomes with cuboctahedral habits comprising {100} and {111} faces (Mann et al., 1984a, b). In other magnetotactic *Alphaproteobacteria*, including magnetotactic cocci and vibrios, the cuboctahedra are elongated parallel to the [111] crystal axis that is oriented parallel to the chain axis. Crystal elongation parallel to [111] results in a non-equidimensional crystal habit with two groups of six {110} faces and two larger and six smaller {111} faces. The six {100} faces remain equidimensional. The result is a prism-like arrangement with a hexagonal cross-section perpendicular to [111] through the center of the crystal (Figure 3) (Towe and Moench, 1981; Meldrum et al., 1993a, b). The remaining faces form corner facets at the intersections between the body {110} and end-cap {111} faces (Figure 3E). The sizes of the crystals, the width/length ratios, and the relative sizes of the corner faces differ between species, resulting in the distinctive projected shapes.

Magnetosomes with elongated-anisotropic habits have been found in three phylogenetic groups of MTB: the *Deltaproteobacteria*, the *Nitrospirae* phylum and the candidate division OP3. The most common 2D projected image of elongated-anisotropic crystals is the bullet or flat-top shape (fts), with one flat end and one narrower, rounded, end (Blakemore et al., 1980; Mann et al., 1987a,b; Thornhill et al., 1994; Isambert et al., 2007) (Figures 4A,B). Sometimes the magnetosome crystals with fts projections are bent in one direction along their length (Hanzlik et al., 2002) (Figure 4B). Some elongated-anisotropic magnetosomes have distinctive projected images with a double-triangle shape (dts), two isosceles triangles sharing a common base (Figure 4C). These dts magnetosomes occur in some MTB phylogenetically affiliated with the *Nitrospirae* and with the *Deltaproteobacteria* (Vali and Kirschvink, 1991; Pósfai et al., 2006; Lins et al., 2007; Li et al., 2010; Lefèvre et al., 2011d). Both projected triangles have the same width, but in mature crystals one triangle is longer than the other.

In MTB of the *Alphaproteobacteria*, magnetosomes arranged in a chain are invariably oriented with a < 111 > crystal axis parallel to the magnetosome chain axis (Mann et al., 1984a,b). In those strains with elongated-prismatic habits, the axis of elongation is the < 111 > axis of orientation (Meldrum et al., 1993a,b) (Table 1). This is not the case for the elongated-anisotropic magnetosomes in MTB affiliated with either the *Nitrospirae* or the *Deltaproteobacteria* (Lefèvre et al., 2011d). While elongated-anisotropic magnetosomes are usually oriented with their long axes parallel to the chain axis, the axis of elongation can vary among the < 100 >, < 110 >, or < 111 > axes (Figures 4, 5). Since the easy magnetization axis in magnetite is parallel to < 111 >, elongation along this direction maximizes the magnetic moment of the crystal because the directions of shape and magnetocrystalline anisotropies coincide. Therefore, the < 111 >-elongations of magnetosomes could be interpreted as selected by evolution. However, no trivial explanation exists for the presence of < 100 > and < 110 >-type elongations which are highly unusual (or maybe even unknown) in inorganic magnetite crystals, and offer no functional advantage for magnetotaxis.

The elongated-anisotropic magnetosomes in the alkaliphilic dissimilatory sulfate-reducing *Deltaproteobacteria*, strain AV-1, and the freshwater *Nitrospirae* strain LO-1 have the following features: (i) the majority of magnetosome crystals have dts projected images and are single crystals without defects or twinning; a minority are strongly curved and comprise several crystallites that are not all in the same orientation; (ii) the habits of the dts magnetosomes can be modeled on the basis of a regular, or slightly elongated, half-octahedron (four-sided pyramid) as a base, and an elongated, pointed section that consists mostly of high-index faces. The curved outlines suggest that the surface of the elongated section cannot be completely described using Miller-indices; (iii) the axis of elongation of the dts magnetosomes is parallel to [100] (Lefèvre et al., 2011d) (Figure 5B).

The bent, elongated-anisotropic, fts magnetite magnetosomes in the moderately thermophilic *Nitrospirae* strain HSMV-1 have the following features: (i) the magnetosomes are highly elongated and many of them are bent in one direction (hook shaped); (ii) from the analysis of high resolution images and their Fourier transforms the principal elongation axis is [110]; (iii) idealized morphological models have elongated “prismatic” side faces that are parallel to [110] and may include certain faces of the {100}, {111}, {110}, and {112} forms (Figure 5A). The narrow, rounded ends of the models consist of faces of the same forms (Lefèvre et al., 2011d). However, it should be noted that these crystals do not appear to be bound by well-developed, smooth, faces and instead, outlines of the crystals are irregular. Thus, any model of these crystals is just an approximation.

## Greigite magnetosome crystals

The first reports on greigite-producing MTB were from samples collected in marine, estuarine, and salt marsh environments (Heywood et al., 1990; Mann et al., 1990). It is only recently that freshwater greigite-producing MTB were described (Lefèvre et al., 2011c; Wang et al., 2013). Only one MTB that synthesizes greigite magnetosomes, *Candidatus* Desulfamplus magnetomortis, is available in pure culture (Lefèvre et al., 2011c). Recognized greigite-producing MTB include the magnetotactic multicellular prokaryotes (MMP) (Farina et al., 1990; Mann et al., 1990) and a variety of relatively large, rod-shaped bacteria (Heywood et al., 1990; Lefèvre et al., 2011c). Like magnetite crystals in magnetosomes, the morphologies of the greigite crystals also appear to be species-and/or strain-specific (Heywood et al., 1991).

While greigite is common in all MTB containing iron sulfide magnetosomes, mackinawite (tetragonal FeS), and tentatively, sphalerite-type cubic FeS were also identified in some (Pósfai et al., 1998a,b). Mackinawite is known to convert to greigite over time under reducing sulfidic conditions (Pósfai et al., 1998b). Orientation relationships between the two minerals indicate that the cubic close-packed S substructure remains unchanged during the transformation; only the Fe atoms rearrange. Planar defects typically occur along the close-packed layers of greigite crystals; such defects indicate that all greigite crystals formed by solid-state transformation from mackinawite or cubic FeS.

In most cases neither the orientations, nor the morphologies of greigite crystals are as strictly controlled as those of magnetite magnetosomes, resulting in fairly disordered chains of irregularly-shaped crystals (Kasama et al., 2006). The habits of greigite magnetosomes are either equidimensional, with irregularly-shaped surfaces that lack clear facets, or slightly elongated parallel to [100]. Since the easy magnetization axis in greigite is thought to be [100] (Hoffmann, 1992), this elongation maximizes the magnetic moment of the crystal. Since all known greigite-producing organisms are affiliated with *Deltaproteobacteria* class, and little information is available on greigite morphologies, further analysis of possible relationships between magnetosome morphologies and the systematics of gregite-producing bacteria appears to be premature.

## Biological control of magnetosome mineralization

The production of magnetite and greigite crystals is under strict genetic control by MTB. The genes encoding the Mam proteins responsible for magnetosome formation are reasonably conserved, with some exceptions, and are located as clusters in close proximity within the genomes of all MTB that have been sequenced (Grünberg et al., 2001; Matsunaga et al., 2005; Jogler and Schüler, 2009; Jogler et al., 2009, 2011; Nakazawa et al., 2009; Schübbe et al., 2009; Abreu et al., 2011; Komeili, 2012; Ji et al., 2013; Lefèvre et al., 2013b). In the genomes of some MTB, the clusters are flanked, and occasionally interrupted, by genomic elements characteristic of a “genomic island” (e.g. transposases, insertion sequences, t-RNA genes); hence the name “magnetosome gene island (MAI)” (Schübbe et al., 2003; Ullrich et al., 2005). The similar organization of the MAI in the genomes of different MTB is the basis for the suggestion that the MAI might have been acquired by different bacterial species via horizontal gene transfer, thereby explaining the great diversity of the group and the apparent polyphyletic trait of magnetotaxis (DeLong et al., 1993). However, recent genomic and phylogenetic studies suggest a monophyletic model in which *mam* genes were acquired by vertical descent from a common ancestor of all MTB (discussed in more detail below) (Lefèvre et al., 2013a).

The minimum set of *mam* genes necessary for magnetosome formation, initially recognized in the *Alphaproteobateria* (Murat et al., 2010, 2012; Lohsse et al., 2011), is also present in the MTB from other phylogenetic affiliations (Lefèvre et al., 2013b). Ten genes (*mamABEIKLMOPQ*) are conserved in all magnetite-producing MTB while only nine of them, excluding *mamL*, appear to be conserved in the two greigite-producing MTB with their genomes sequenced (Abreu et al., 2011; Lefèvre et al., 2013b). In addition to this core of *mam* genes, other genes are present in the vicinity of the chromosomal region containing magnetosome genes. The *mms* genes and the *mamXY* and *mamGFDC* clusters are specific to the magnetotactic *Alphaproteobacteria*, whereas the *mad* genes are specific to and conserved within the *Deltaproteobacteria* class and the *Nitrospirae* phylum (Lefèvre et al., 2013b). Thus, despite the nine *mam* genes that seem to be absolutely necessary for the formation of magnetite and greigite magnetosomes (vesicle formation, iron uptake, nucleation of the crystal and alignment of magnetosomes) other genes are also MTB-specific but only present in certain groups. These genes are likely involved in control of the crystal size, morphology and organization of the magnetosomes or potential functions related to magnetotaxis (e.g. aerotaxis) (Scheffel et al., 2008; Murat et al., 2010; Lohsse et al., 2011; Lefèvre et al., 2013b).

## Phylogenetic significance

**Figure 6:**
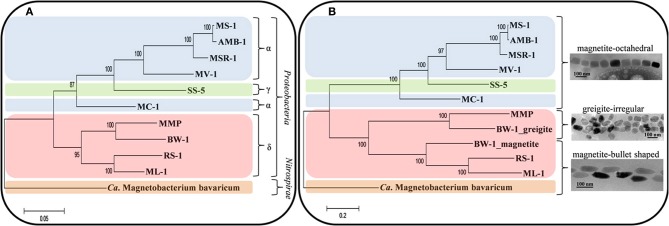
**Evolution and transfer of magnetosomes**. Phylogenetic trees based on 16S rRNA gene sequences reflecting the evolution of MTB **(A)** and on concatenated magnetosome protein sequences (MamABEIKMPQ and FeoB) reflecting the evolution of magnetotaxis **(B)**. Reproduced with permission from Lefèvre et al. (2013a,b). Trees were constructed applying the maximum likelihood algorithm. Bootstrap values at nodes were calculated with 100 replicates. Magnetotactic strains used for the analysis are *Magnetospirillum magnetotacticum* (MS-1), *Ms*. *magneticum* (AMB-1), *Ms*. *gryphiswaldense* (MSR-1), *Magnetovibrio blakemorei* (MV-1), *Magnetococcus marinus* (MC-1), strain SS-5, the magnetotactic multicellular prokaryote “*Candidatus* Magnetoglobus multicellularis” (MMP), “*Ca*. Desulfamplus magnetomortis” (BW-1), *Desulfovibrio magneticus* (RS-1), strain ML-1, and “*Ca*. Magnetobacterium bavaricum.”

If the magnetotactic trait was transferred between the different phylogenetic groups that contain MTB through horizontal gene transfer, we would expect to find all magnetosome crystal morphologies in the different groups of MTB. Indeed, the genetic information responsible for mineral type and morphology, although originating from a similar core of Mam proteins, is sufficiently variable to retrace the evolutionary history of the different morphological types. Recently, it was shown that phylogenetic trees based on Mam proteins, reflecting the evolution of magnetosomes, and the tree based on the 16S rRNA gene sequences, reflecting the evolution of MTB, are congruent (Lefèvre et al., 2013a) (Figure 6). This indicates that all MTB evolved from a common ancestor with magnetosome genes. Since the magnetosome genes appear to have been mainly transferred by descent with the accrual of variations over time, it is logical to expect a similar pattern of evolution between the 16S rRNA genes, the Mam proteins and the type of magnetosomes in the different species or strains of MTB (Figure 6). This new model of transfer of magnetosome formation by descent from a common ancestor to all MTB suggests that in the past all bacteria, magnetotactic or not, sharing a common ancestor with MTB (i.e., all the *Proteobacteria* and likely all the *Nitrospirae* and OP3 division) were capable of magnetosome formation, but many lost this capacity over time. Indeed, during evolution, *Proteobacteria* and *Nitrospirae* diverged greatly in their ecophysiology. Thus, magnetosome formation likely became obsolete in most microorganisms of these phyla and the magnetotactic trait was lost through the loss of the magnetosome genes (Lefèvre and Wu, 2013).

As previously noted, magnetotactic *Alpha-* and *Gammaproteobacteria*, the later diverging classes of the *Proteobacteria*, biomineralize magnetite that include cuboctahedral and elongated prisms (Devouard et al., 1998; Lefèvre et al., 2012) (Figures 1–3). All the elongations are parallel to < 111 >. In contrast, in the magnetotactic *Deltaproteobacteria*, the most deeply diverging group of the *Proteobacteria* that biomineralize magnetite or greigite or both, the magnetite crystals are always bullet-shaped (Figure 4C). Greigite-producing MTB form a monophyletic clade in the *Deltaproteobacteria* class (Lefèvre et al., 2013a). The magnetotactic *Nitrospirae* and strain SKK-01 of the candidate division OP3, the most deeply branching phylogenetic groups that contain MTB (Jogler et al., 2011; Kolinko et al., 2012), are known to biomineralize magnetite crystals whose morphologies are very similar, if not identical, to those found in the *Deltaproteobacteria* (Figure 4B) (Lefèvre et al., 2011d). Thus, there is a strong and important correlation between the morphology and the composition of the magnetosomes produced by MTB and their phylogenetic affiliation (Abreu et al., 2011; Jogler et al., 2011; Kolinko et al., 2012; Lefèvre et al., 2012, 2013a). Because MTB in the most deeply branching phylogenetic groups all have magnetite magnetosomes with similar, elongated-anisotropic habits, it has been suggested that the earliest magnetosome mineral phase in MTB was elongated-anisotropic magnetite (Lefèvre et al., 2013a).

The recent sequencing of the genomes of two greigite-bearing bacteria, *Ca*. Magnetoglobus multicellularis (Abreu et al., 2011) and *Ca*. Desulfamplus magnetomortis (Lefèvre et al., 2011c), gave genomic and phylogenetic evidence that the gene cluster responsible for greigite production emerged from duplication and successive rearrangement of the gene cluster responsible for magnetite formation (Lefèvre et al., 2013b). This duplication likely led to the adaptation of MTB to highly reduced environments. Indeed, greigite producers are generally found in reduced environments with high concentrations of hydrogen sulfide in the sediments (Bazylinski et al., 1995; Lefèvre et al., 2011c). *Ca*. Desulfamplus magnetomortis, the only greigite producer in axenic culture that also produces magnetite, has two clusters of *mam* genes in its genome; one presumably involved in magnetite formation and the other in greigite formation. There is evidence that depending on environmental conditions, this bacterium preferentially mineralizes magnetite (high redox potential) or greigite (reduced conditions) (Lefèvre et al., 2011c, 2013b). In environmental studies of chemically-stratified coastal ponds, it was found that magnetite producers are mostly found at the oxic-anoxic interface (OAI) while greigite producers are found below the OAI, in a more reduced biotope (Bazylinski et al., 1995; Simmons et al., 2004). Thus, even if there is genetic control over the type of crystal mineralized in the magnetosome membrane, environmental parameters also play a role in the regulation of the chemical composition of the crystal. Since octahedral and anisotropic magnetite particles can be formed in microaerobic as well as in anaerobic conditions, it is not known if specific environmental conditions are required for their morphological differentiation.

## Magnetosomes in eukaryotes?

A number of eukaryotes also appear to use magnetic fields for orientation, navigation, and homing, a process known as magnetoreception (Kirschvink et al., 2001). In some cases, magnetoreception appears to be due to the presence of magnetosomes or magnetosome-like structures that contain single magnetic domain crystals of magnetite as do most known MTB. These organisms include single-celled eukaryotes such as algae and protists (Torres de Araujo et al., 1986; Bazylinski et al., 2000, 2012) and higher organisms such as the sockeye salmon, *Oncorhynchus nerka* (Mann et al., 1988) or the honey bee, *Apis mellifera* (Kuterbach et al., 1982). A key question regarding these organisms is how they obtained and built these structures.

The alga, discovered in brackish mud and water samples collected from a coastal mangrove swamp near Fortaleza, Brazil, was tentatively identified as *Anisonema platysomum* and exhibited magnetotaxis (Torres de Araujo et al., 1986). Cells contain numerous, well-organized chains of bullet-shaped magnetite crystals. Other magnetotactic protists have since been shown to contain magnetite crystals (Bazylinski et al., 2000). Thus, the origin of these putative “magnetosomes” in magnetotactic protists is an important question (Bazylinski et al., 2012). There appear to be two possibilities: the protists biomineralize the magnetite crystals themselves or they ingest MTB and/or bacterial magnetosomes from lysed MTB cells and incorporate them either temporarily or permanently in the cell. Both scenarios seem to occur in nature. The arrangement of magnetosomes appears to be so precisely structured in the euglenoid alga described by Torres de Araujo et al. (1986), it seems likely that this organism biomineralizes and arranges endogenous magnetite crystals in a highly controlled fashion within the cell, where intracellular structural filaments play a significant role in the synthesis of the magnetosome chain, as has been shown for MTB (Komeili et al., 2006; Scheffel et al., 2006). For this arrangement to occur by ingesting MTB, significant numbers of MTB would have to be consumed and because the magnetite crystals are all bullet-shaped, the MTB would all have to be from specific phylogenetic groups (e.g. *Deltaproteobacteria*), which seems implausible. Other magnetotactic protists, including dinoflagellates, biflagellates, and ciliates, contain magnetosomes that are not well-organized in the cell and thus probably ingest MTB and contain the bacterial magnetosomes for an undetermined amount of time (Bazylinski et al., 2000, 2012). The specific crystal habits of these latter protists have not been examined.

Cells of the ethmoid tissue of *Oncorhynchus nerka* contain chains of well-ordered crystals of cuboctahedral crystals of magnetite with {111} faces of adjacent crystals lying perpendicular to the chain axis (Mann et al., 1988). The consistent structural features of these particles suggest that they are biomineralized by the organism as in the magnetotactic alga. If we assume that the magnetite crystals in both the alga and *O. nerka* are biomineralized by the organism and address similar questions that we discuss about biomineralization of magnetosomes in MTB, an obvious question, amongst many others, is how these eukaryotic organisms control the size and morphology of their magnetite crystals? Do these organisms contain similar genes to the magnetosome (e.g. *mam*) genes of MTB? If so, will the genes of the alga be more similar to those of the phylogenetic groups of MTB that biomineralize bullet-shaped magnetosome magnetite crystals and those of *O. nerka* more similar to those of MTB that also produce cuboctahedral magnetosome magnetite particles? How do these findings relate to the evolution of the magnetosome? One intriguing idea discussed a number of years ago was the possibility that an MTB was the ancestral eukaryotic host cell (Vali and Kirschvink, 1991; Kirschvink and Hagadorn, 2000). However, no genes orthologous to Mam, Mms or Mad genes were found in the available genomes of eukaryotes able to produce intracellular magnetosome-like structures (unpublished data). It is possible that the genes responsible for magnetosome formation in prokaryotes and eukaryotes may have diverged too greatly to be recognized as orthologous. It is also possible that magnetosome formation in prokaryotes and eukaryotes has had separate origins, i.e., magnetosome formation in these two domains is polyphyletic. It is clear that magnetosome formation in eukaryotes is much less understood than in prokaryotes.

## Conclusions and perspectives

We have shown in this review that the morphological properties of magnetosome minerals correlate strongly with specific phylogenetic groups of MTB thus reflecting the evolutionary path of magnetotaxis. While a number of genes are clearly important in the biomineralization process, those genes responsible for magnetosome crystal morphology are not yet known. Nonetheless, phylogenetic analyses of magnetosome proteins indicate that the first magnetosomes contained bullet-shaped crystals of magnetite (Lefèvre et al., 2013a).

In addition, it is known that a number of environmental parameters influence the morphology and composition of the magnetosome crystals, although they have been little studied. Despite the fact that reducing, sulfidic environments appear to favor the formation of greigite (Bazylinski et al., 1995; Simmons et al., 2004; Lefèvre et al., 2011c), and that rates of iron uptake by MTB appear to change the morphology of magnetite crystals (Faivre et al., 2008), we do not know what chemical or physical parameters regulate magnetosome formation and control their morphology. Nevertheless, the environmental factors appear to have only slight control over magnetosome morphologies, and the basic crystal habits (cuboctahedral, elongated-prismatic, and elongated-anisotropic) are clearly determined by genetics.

It is also important to note that magnetite and greigite formation continue to occur under anaerobic conditions (where MTB respire with nitrate, nitrous oxide or sulfate as terminal electron acceptors), where chemical or redox gradients are absent and chemical conditions are homogeneous. Here magnetotaxis does not provide an apparent advantage. This suggests that magnetosomes may have additional functions in the absence of oxygen.

Progress in these domains will require the development of new genetic systems in all taxa in which MTB occur, additional genomic studies of new MTB and highly controlled growth studies in which the effects of specific environmental parameters can be precisely determined. Understanding how eukaryotes biomineralize magnetosome-like structures will require initiation of molecular and genomic studies.

### Conflict of interest statement

The authors declare that the research was conducted in the absence of any commercial or financial relationships that could be construed as a potential conflict of interest.
